# The MK2/3 cascade regulates AMPAR trafficking and cognitive flexibility

**DOI:** 10.1038/ncomms5701

**Published:** 2014-08-19

**Authors:** Katherine L. Eales, Oleg Palygin, Thomas O’Loughlin, Seyed Rasooli-Nejad, Matthias Gaestel, Jürgen Müller, Dawn R. Collins, Yuriy Pankratov, Sonia A.L. Corrêa

**Affiliations:** 1 School of Life Sciences, University of Warwick, Coventry CV4 7AL, UK; 2 Institute of Biochemistry, Hannover Medical University, 30625 Hannover, Germany; 3 Warwick Medical School, University of Warwick, Coventry CV4 7AL, UK; 4 School of Life Sciences, University of Bradford, Bradford BD7 1DP, UK

## Abstract

The interplay between long-term potentiation and long-term depression (LTD) is thought to be involved in learning and memory formation. One form of LTD expressed in the hippocampus is initiated by the activation of the group 1 metabotropic glutamate receptors (mGluRs). Importantly, mGluRs have been shown to be critical for acquisition of new memories and for reversal learning, processes that are thought to be crucial for cognitive flexibility. Here we provide evidence that MAPK-activated protein kinases 2 and 3 (MK2/3) regulate neuronal spine morphology, synaptic transmission and plasticity. Furthermore, mGluR-LTD is impaired in the hippocampus of MK2/3 double knockout (DKO) mice, an observation that is mirrored by deficits in endocytosis of GluA1 subunits. Consistent with compromised mGluR-LTD, MK2/3 DKO mice have distinctive deficits in hippocampal-dependent spatial reversal learning. These novel findings demonstrate that the MK2/3 cascade plays a strategic role in controlling synaptic plasticity and cognition.

## Abstract (web summary)

MK2/3 kinases are both highly expressed in the hippocampus and cortex of the brain, but their role is unclear. Here Eales *et al.* show that MK2/3 signalling regulates dendritic spine morphology and is required for synaptic plasticity via the regulation of AMPA receptor trafficking, and cognitive processes.

## Graphical abstract

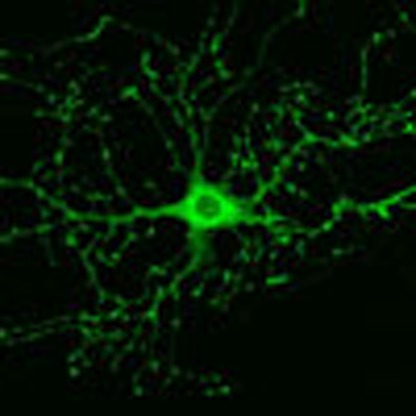

Modulation of glutamatergic synaptic transmission is termed synaptic plasticity and can last for days. These long-lasting alterations in glutamatergic transmission may provide the molecular substrate that underlies learning and memory. Long-term depression (LTD) is the sustained decrease in synaptic transmission and involves either activation of ionotropic NMDA (*N*-methyl-D-aspartate) receptors (NMDAR-dependent LTD) or metabotropic glutamate receptors (mGluR-dependent LTD)^1^,^2^. Over the past decade, several studies have shed light on the molecular mechanisms and signalling pathways that trigger and maintain mGluR-LTD and there is strong evidence for direct links between neuronal dendritic spine shrinkage, endocytosis of glutamatergic α-amino-3-hydroxy-5-methyl-4-isoxazole propionic acid receptors (AMPAR) located on the spine surface and decreases in miniature-excitatory post-synaptic currents (mEPSC) in mGluR-dependent LTD^1^,^2^,^3^,^4^. Furthermore, there is correlation between actin reorganization and AMPAR endocytosis following induction of mGluR-dependent LTD^4^,^5^,^6^. At dendritic spines, actin is found in either monomeric globular (G)-actin or filamentous (F)-actin forms, the dynamic shift between these two arrangements promotes actin remodelling and subsequent changes in spine morphology^4^. However, the steps linking activation of mGluR to changes in spine morphology and reductions in synaptic transmission promoted by AMPAR endocytosis underlying LTD remains elusive.

A pathway well-placed to provide this link is the p38 signalling cascade, which is required for mGluR-LTD^7^,^8^; however, the downstream targets are not yet characterized. The MAPK-activated protein kinases 2 and 3 (MK2/3) serine/threonine kinases are strong candidates, as they bind and are activated by p38α/β isoforms. Interestingly, p38α/β isoforms and MK2/3 kinases are both highly expressed in the brain, particularly the hippocampus and cortex^8^,^9^,^10^. Although MK2 and MK3 are structurally related proteins, the interaction between p38α and MK2 is best characterized. Once activated, p38α binds and phosphorylates MK2 causing a conformational change and allowing the active MK2-p38α complex to phosphorylate its substrates^11^,^12^. Although little is known about MK2/3’s physiological function in the brain, or the involvement of the p38-MK2/3 cascade in actin reorganization^11^, it has been shown that vascular endothelial growth factor-A induces actin reorganization through activation of the p38-MK2-LIM kinase1-cofilin1 cascade^13^. Cofilin1 is a well-characterized protein recognized mainly for its role in depolymerization of actin filaments^4^. The importance of cofilin1-regulated actin remodelling in synaptic plasticity is well documented^14^, however, the signalling pathways regulating this process remains elusive.

Here we used MK2/3 double knockout (DKO) mice to show that the MK2/3 cascade regulates spine morphology, trafficking of AMPAR and glutamatergic synaptic transmission in hippocampal neurons. Under basal conditions, absence of MK2/3 in cultured hippocampal neurons results in dramatic changes in spine morphology, altered levels of cofilin1 activity and decreased AMPAR-mediated mEPSC amplitude. Furthermore, mirroring the abnormal spine formation, MK2/3 DKO hippocampal neurons have deficits in GluA1 endocytosis and mGluR-LTD. Consistent with compromised mGluR-LTD, DKO mice have distinctive deficits in hippocampal-dependent spatial reversal learning. The data presented here provide compelling evidence that the p38-MK2/3 cascade is a mechanistic link between the mGluR-activation and AMPAR endocytosis underlying mGluR-dependent LTD. These findings identify the p38-MK2/3 cascade as a potential therapeutic target for slowing cognitive decline observed in natural ageing.

## Results

### MK2 regulates spine morphology and synaptic transmission

**Figure 1:**
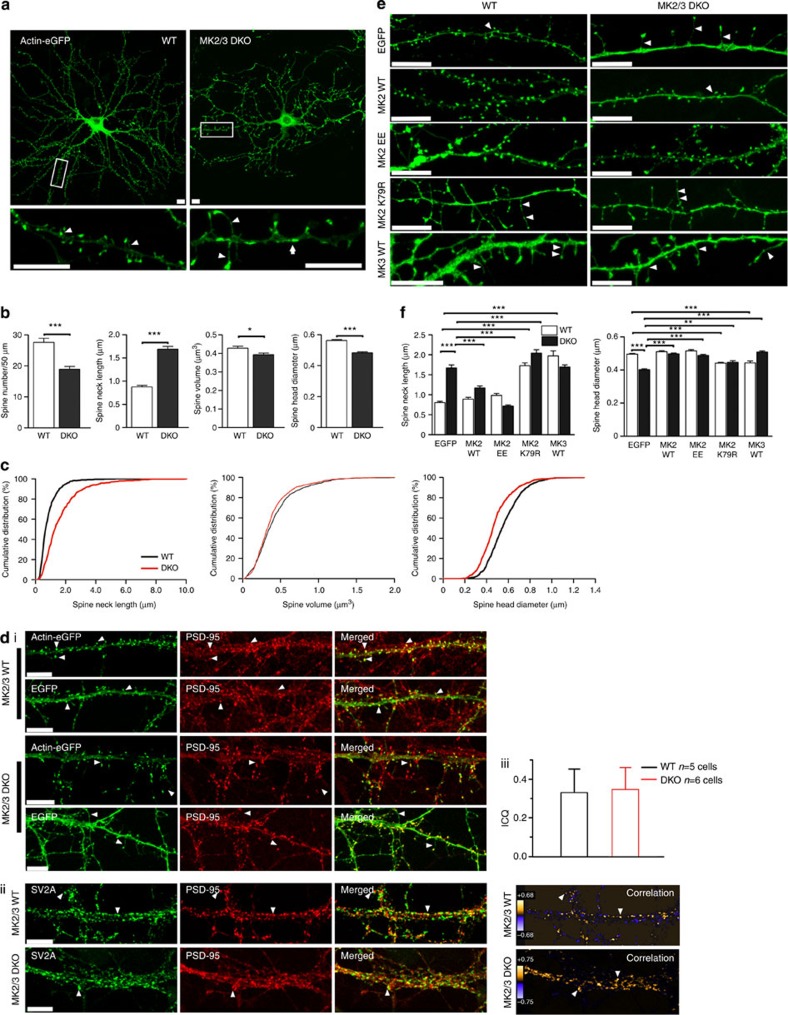
MK2 regulates spine morphology in hippocampal neurons. (**a**) Images of cells expressing actin-EGFP. Note that actin accumulates at spine heads in WT cells, whereas in DKO cells actin accumulates along the process (arrows) and the spines display longer neck and smaller head diameters (arrowheads). (**b**) Graphs showing decreased spine density per 50 μm (WT: 27.6±1.34, 29 processes; DKO: 18.9±0.91; 28 processes), volume (μm^3^; WT: 0.43±0.01; 796 spines, 17 cells; DKO: 0.39±0.01; 801 spines, 23 cells), head diameter (μm; WT: 0.56±0.01; 796 spines, 17 cells; DKO: 0.48±0.01; 801 spines, 23 cells) but increased neck length (μm; WT: 0.88±0.03; 472 spines, 29 processes; DKO: 1.69±0.06; 464 spines, 28 processes). (**c**) Cumulative distribution of data shown in **b**. Note that the majority of the spines in DKO cells display longer necks compared with WT (***P<0.001), smaller spine head diameter (***P<0.001) and volume (**P<0.01). (**d**(i)) WT and DKO cells expressing EGFP or actin-EGFP co-stained with PSD95. Note the co-localization of the long and thin protrusions with PSD95 in DKO cells (arrowheads). (ii) Non-transfected cells co-stained with SV2a and PSD95. (iii) Co-localization analysis of pre- and postsynaptic marker. (**e**) WT and DKO neurons expressing a range of MK2- and MK3-EGFP-tagged constructs. (**f**) WT cells expressing MK2-K79R shows increased spine neck length (EGFP: 0.81±0.03, 412 spines; MK2-WT: 0.89±0.05, 189 spines; MK2-EE: 0.99±0.05, 385 spines; MK2-K79R: 1.73±0.07, 454 spines) and reduced head diameter (EGFP: 0.50±0.01, 629 spines; MK2-WT: 0.51±0.01, 672 spines; MK2-EE: 0.52±0.01, 564 spines; MK2-K79R: 0.44±0.01, 727 spines). Interestingly, spine neck length was reduced to WT levels in DKO cells expressing MK2-WT and MK2-EE, but not in cells expressing MK3-WT (EGFP: 1.67±0.08, 220 spines; MK2-WT: 1.17±0.05, 367 spines; MK2-EE: 0.72±0.03, 257 spines; MK2-K79R: 2.05±0.09, 296 spines; MK3-WT: 1.694±0.05, 700 spines), and head diameter was increased in DKO cells expressing MK2-WT, MK2-EE, MK2-K79R and MK3-WT (EGFP: 0.40±0.01 μm, 468 spines; MK2-WT: 0.50±0.01 μm, 909 spines; MK2-EE: 0.49±0.01 μm, 709 spines; MK2-K79R: 0.45±0.01 μm, 370 spines; MK3-WT: 0.509±0.01μm, 700 spines). An unpaired (Mann–Whitney) *T*-test (**b**), Kolmogorov–Smirnov (**c**) and one-way analysis of variance (**d**) with appropriate *post-hoc* test was used. Error bars=±s.e.m., *P<0.05, **P<0.01, ***P<0.001. Scale bar, 10 μm.

Considering that p38 is required for mGluR-LTD, a process requiring actin reorganization^5^,^6^,^15^, and that p38-MK2 regulates actin remodelling through distinct pathways^13^,^16^, we asked whether the absence of MK2/3 affected spine morphology. First, we transfected hippocampal cultures from wild-type (WT) and DKO mice with an actin-enhanced green fluorescent protein (eGFP) construct. Actin accumulates mainly at the spines, enabling precise visualization and analysis of spine shape and density. Morphological analysis showed significant reductions in spine density and significant increases in spine neck length in DKO neurons compared with WT (Fig. 1a,b). Strikingly, the average neck length in DKO was nearly double that in WT neurons (Fig. 1a,b). Cumulative distribution showed that the majority of DKO spines display these abnormalities (Fig. 1c). Confocal images showed that WT spines were bulbous and stubby, with actin accumulating mainly in the spine head, whereas DKO spines had an elongated shape, with actin accumulating not only at the spine head, but surprisingly also in the processes (Fig. 1a). To confirm that these long, thin protrusions were indeed spines, we showed that the actin-transfected spines co-localized with the postsynaptic marker PSD95 (Fig. 1d(i)). Furthermore, there is good correlation between levels of co-localization of pre- and postsynaptic markers SV2a and PSD95 in non-transfected neurons from WT and DKO mice (Fig. 1d(ii,iii)). This observation suggests that the absence of MK2 and MK3 does not have a strong effect on apposition of pre- and postsynaptic sites.

We next considered whether MK2/3 promoted the morphological changes seen in DKO cells. To address this, we overexpressed catalytically active WT MK2 (pEGFP-C1-MK2-WT) and MK3 (pEGFP-C1-MK3-WT) in WT and DKO cells. The corresponding vector expressing EGFP alone acted as the control for transfection. To show that EGFP-tagged constructs were expressed at spines and could reliably be used as a morphological marker, we co-stained EGFP-transfected cells with the postsynaptic marker, PSD95 (Fig. 1d). No difference in spine density, spine neck length or spine head diameter was observed in WT cells expressing the MK2 construct when compared with cells expressing EGFP alone (Fig. 1e,f). The MK2 construct was then used to transfect MK2/3 DKO cells to see if the striking phenotype observed in these cells could be rescued; average spine neck length and head diameter of DKO cells expressing MK2 was indeed comparable the values seen in WT (Fig. 1e,f). Interestingly, MK3 re-insertion in DKO cells did not rescue the phenotype observed in DKO cells (Fig. 1e,f).

As MK2 insertion into DKO cells rescued the morphological deficits, we considered whether re-insertion of a catalytic-dead MK2 mutant, where a mutation in conservative lysine (K)79 of the ATP-binding subdomain II was replaced with an arginine (pEGFP-C1-MK2-K79R) and a constitutively active MK2 mutant, where the two sites necessary to activate MK2, Thr205 and Thr317, were mutated to glutamic acid (pEGFP-C1-MK2-EE)^17^,^18^ in WT and DKO cells would promote any changes in spine morphology. Re-insertion of MK2-EE did not promote changes in WT cells, however, re-insertion of the kinase-dead MK2-K79R in WT cells caused a dramatic increase in spine neck length (similar to values observed in DKO cells; Fig. 1e,f). The MK2 rescue effect was enhanced when DKO cells were transfected with constitutively active MK2-EE. Interestingly, not only was spine neck length reduced to lengths comparable to WT cells, but also a significant increase in spine density was observed when compared with DKO cells expressing EGFP alone. Importantly, DKO cells overexpressing MK2-K79R displayed even longer necks than DKO-expressing EGFP alone. Spine head diameter was also analysed after transfection with MK2 constructs. DKO cells overexpressing MK2-WT and MK2-EE constructs displayed spine head diameters similar to those in WT cells. Conversely, when WT cells were transfected with MK2-K79R, there was a significant decrease in spine head diameter compared with WT. Surprisingly, MK3-WT expression in WT promoted morphological deficits comparable to those in WT cells expressing the MK2-K79R construct (Fig. 1e,f). This may suggest that MK3 functions as a dominant negative. Together these findings suggest that removal of MK2/3 kinase activity promotes the striking, abnormal phenotype observed in DKO spines.

**Figure 2:**
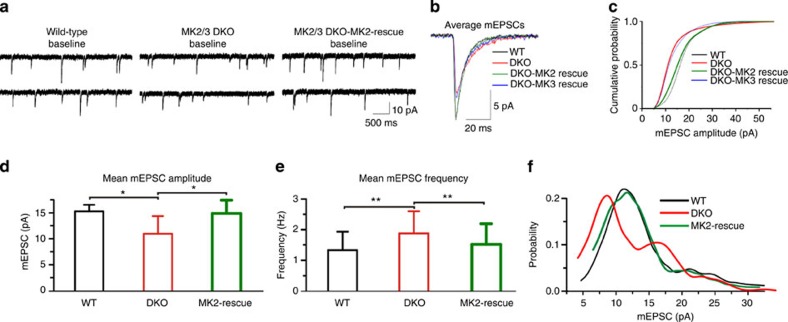
MK2 regulates synaptic transmission in hippocampal neurons. (**a**) AMPAR-dependent mEPSC traces from WT, DKO and DKO neurons expressing EGFP-tagged-MK2-WT (rescue) at baseline. (**b**) AMPAR-dependent mEPSC events recorded from WT, DKO and DKO neurons expressing MK2-WT and MK3-WT. (**c**) Cumulative distribution of all mEPSC amplitudes recorded. (**d**) Mean mEPSC amplitude and (**e**) frequency showing that the decrease in amplitude and increase in frequency observed in DKO are reversed by re-insertion of MK2-WT. (**f**) Note that the double peaks in mEPSC amplitude distribution in DKO cells, suggesting multiquantal release, are not seen in WT nor in DKO expressing MK2-WT. Shown are data from WT (*n*=8 cells), DKO (*n*=10 cells) and DKO overexpressing MK2-WT (*n*=6 cells) from three to four independent preparations. Error bars represent ±s.e.m. and *P<0.04; **P<0.01.

To test whether DKO cells showed deficits in basal synaptic transmission, we recorded spontaneous mEPSC from WT, DKO and DKO hippocampal neurons overexpressing either MK2-WT or MK3-WT. A significant decrease in mean AMPAR-dependent mEPSC amplitude and moderate increase in baseline frequency was observed in DKO neurons compared with WT (Fig. 2a–f). However, these changes were completely restored to WT levels in DKO neurons overexpressing MK2-WT. Expression of MK3-WT in DKO cells had no significant effect on mEPSC amplitude (Fig. 1b,c). These data suggest that the reduced mEPSC amplitude and increased mEPSC frequency observed in DKO cells may result from a reduction in number of AMPAR subunits present postsynaptically, concomitant with increases in glutamate release from presynaptic terminal. However, another plausible explanation is that reduced levels of surface AMPAR in DKO cultures promotes an increased frequency of mEPSC to compensate for lower activity at the postsynaptic membrane^19^,^20^. Interestingly, analysis of release probability (Fig. 2f) showed a clear double peak in DKO cells instead of the single peak seen in WT cells and in MK2/3 DKO cells expressing MK2-WT. We therefore speculate that reduced activity at the postsynaptic membrane in DKO leads to multi-vesicle release, maintaining synapses at the optimal operational range^21^. Together, these data show that absence of MK2/3 promotes abnormalities in spine morphology and deficits in synaptic transmission in hippocampal neurons. Furthermore, these changes are reversed when MK2, but not MK3, is re-introduced into DKO cells, suggesting that loss of MK2 is responsible for the deficits in glutamatergic synaptic transmission.

### mGluR-LTD is impaired in MK2/3 DKO hippocampal neurons

**Figure 3:**
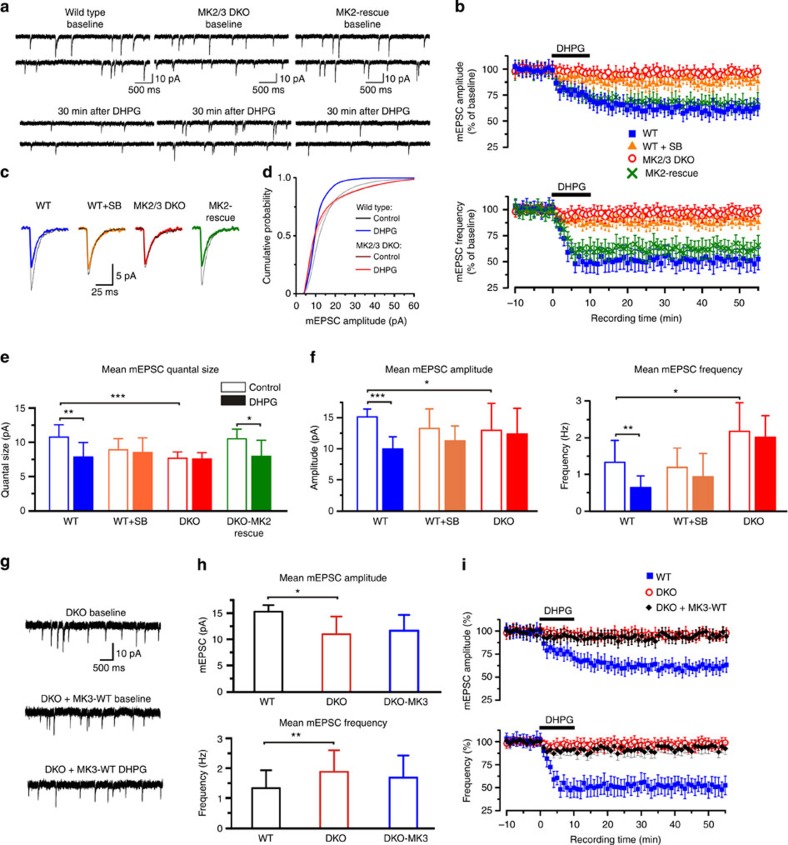
mGluR-LTD is impaired in MK2/3 DKO hippocampal neurons. (**a**) AMPAR-dependent mEPSC traces from WT, DKO and DKO neurons expressing eGFP-tagged-MK2-WT (rescue) at baseline and 30 min after DHPG exposure. (**b**) Graphs showing average time course of changes in mEPSC amplitude and frequency. Average mEPSC amplitude was 60.6±7.4% of baseline at 45±5 min (blue trace; *n*=8) in WT, 87.9±6.3% in WT cells pre-incubated with the p38 inhibitor SB 203580 (5 μM; orange trace; *n*=7) and 95.7±7.2% (red trace; *n*=10) in DKO. Note that LTD was rescued in DKO expressing MK2-WT 66.5±7.7% (green trace; *n*=6). Average mEPSC frequency of WT cells was 51.9±8.9 of the baseline at 45±5 min following LTD induction (blue), compared with 88.3±5.8 in WT cells pre-incubated with p38 inhibitor (orange) and to 96.7±7.2 in DKO cells (red). Re-insertion of MK2-WT in DKO cells rescued the frequency (63.2±8.5% of baseline; green) to WT values. (**c**) Example of mEPSC waveforms recorded during baseline (black traces) and after DHPG exposure (coloured traces) for the cell recorded in **a**. (**d**) Cumulative mEPSC amplitude distributions from WT and DKO cells. (**e**) Graphs of mean mEPSC quantal size for all experimental conditions. Note that the DHPG-dependent reduction in activity observed in wild-type cells (blue bars) is occluded in DKO cells (red bars) but restored in DKO cells expressing MK2-WT protein. (**f**) Graphs showing that the DHPG-dependent reduction in frequency and amplitude observed in WT cells are impaired in DKO cells. (**g**) Representative mEPSC waveforms recorded from DKO; DKO cells expressing MK3-WT at baseline and 30 min after DHPG exposure. (**h**) Graph of mean mEPSC amplitude (DKO: 10.9±3.4 pA and DKO+MK3-WT: 11.7±3.5 pA) and frequency (DKO: 1.85±0.72 Hz and DKO+MK3-WT: 1.71±0.74 Hz) at baseline and (**i**) after DHPG exposure. Data shown are from DKO (*n*=14 cells); DKO cells expressing MK3-WT at baseline (*n*=6 cells) and after DHPG exposure (*n*=4 cells). Error bars=±s.e.m. Recordings were done in the presence of tetrodotoxin (1 μM), picrotoxin (50 μM) and L689–560 (5 μM). Error bars indicate the ±s.e.m. and *P<0.02, **P<0.01, ***P<0.005.

Considering that p38 is required for mGluR-LTD^15^ and that MK2/3 DKO neurons displayed reduced spine head diameter and longer spine neck length, morphological characteristics comparable to those observed in WT neurons following induction of mGluR-LTD^5^,^8^, we then asked whether the p38-MK2/3 cascade regulates mGluR-LTD. In agreement with previous studies, (RS)-3,5-dihydroxyphenylglycine (DHPG) application (100 μM, 10 min) to WT neurons resulted in a persistent decrease in the frequency^22^,^23^, amplitude and quantal size of mEPSC (Fig. 3a–f). It is important to note that with decreased synaptic transmission, significant reductions in mEPSC amplitude (that is, postsynaptic effects) can result in events occurring below the threshold of detection, which can be misinterpreted as reduced frequency (that is, presynaptic effects). To avoid this, we combined methods developed to analyse small amplitude mEPSC^24^ with manual analysis. This allowed us to reliably detect synaptic currents of reduced amplitude and evaluate changes in both quantal size and frequency of mEPSC.

To investigate the role of p38 in DHPG-LTD, WT cells were pre-incubated with the p38 inhibitor SB 203580 (SB, 5 μM), before DHPG application. SB blocked the reduction of mEPSC amplitude and frequency observed in DHPG-LTD (Fig. 3b–f). Furthermore, DHPG failed to induce significant decreases in mEPSC frequency and amplitude in DKO cells (Fig. 3d). As insertion of MK2-WT cDNA in DKO cells rescued the deficits in spine morphology (Fig. 1), we asked whether re-insertion of MK2-WT in MK2/3 DKO cells would restore mGluR-LTD. Indeed, DHPG-LTD was completely rescued in DKO cells overexpressing MK2-WT (Fig. 3a-e), showing that MK2 activity is sufficient to induce mGluR-LTD. DHPG exposure failed to promote DHPG-dependent LTD in DKO-expressing MK3-WT (Fig. 3g–i).

**Figure 4:**
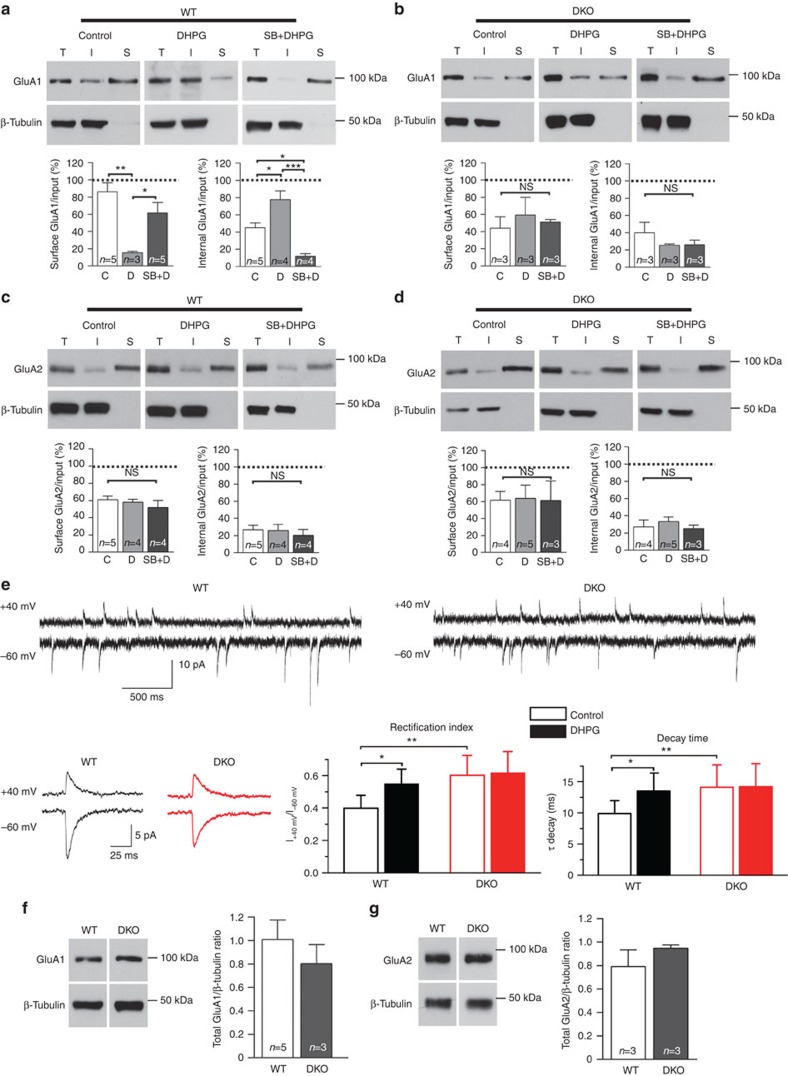
AMPAR trafficking is disrupted in MK2/3 DKO neurons. (**a**) The amount of GluA1 at the surface (S) and internal (I) were analysed in WT hippocampal cultures in control (C), DHPG (D; 100 μM) stimulated and cells pre-incubated with SB 203580 (5 μM; SB+DHPG). Blot band analyses show significant decreases in surface GluA1 (control: 86.3±10.1%; DHPG: 15.5±1.4%; SB+DHPG: 61.3±12.4%) and significant increases in internal (I) GluA1 (control: 45.3±5.3%; DHPG: 77.6±10.1%; SB+DHPG: 11.8±3.3%) after DHPG application. Note that pre-incubation with SB prior DHPG exposure blocked the accumulation of internal GluA1. (**b**) GluA1 endocytosis is blocked in DKO cultures treated with DHPG. Blot analysis showing that DHPG exposure did not cause significant changes in the amount of GluA1 at the surface (control: 44.0±13.4%; DHPG: 56.2±20.8%; SB+DHPG: 51.2±2.7%) or in the amount of internal (I) GluA1 (control: 39.8±12.2%; DHPG: 25.4±1.5%; SB+DHPG: 26.0±5.1%). (**c**,**d**) Blot analysis showing that DHPG exposure did not cause any significant changes in the amount of GluA2 at the surface in WT (control: 60.93±4.3%; DHPG: 58.13±3.36%; SB+DHPG: 51.82±8.3%) or in DKO cells (control: 61.54±10.55%; DHPG: 63.96±15.43%; SB+DHPG: 61.20±22.91%). Data were obtained from at least three independent preparations. A one-way analysis of variance and appropriate *post-hoc* test was conducted. Error bars=±s.e.m. and *P<0.05, **P<0.01, ***P<0.001. (**e**) Representative mEPSC traces from WT and DKO neurons recorded at +40 mV and −60 mV holding potentials in the presence of spermine (100 μM) in the intracellular solution. Note the clear reduction in the peak amplitude and increase in decay time of the mEPSC waveform of DKO cell compared with WT held at −60 mV (average of events over 1 min). The rectification index and the constant decay were calculated by dividing the peak amplitude values and the decay time at +40 mV by −60 mV. For rectification index, WT (*n*=6 cells; control: 0.398±0.081, DHPG: 0.549±0.092) and DKO (*n*=5 cells; control: 0.602±0.123, DHPG: 0.618±0.132). For decay constant, WT (*n*=9 cells; control: 9.89±2.1, DHPG: 13.5±2.9) and DKO (*n*=10 cells; control: 14.1±3.6, DHPG: 14.2±3.7). Error bars indicate±s.d. and *P<0.05, **P<0.01. (**f**) Blot analysis showing GluA1 and GluA2 expression levels in WT and DKO cells. β-Tubulin was used as loading control. NS, not significant.

Several studies report that mGluR-LTD is dependent on endocytosis of AMPAR subunits GluA1 and GluA2 (refs 1, 2, 3, 22). We therefore investigated whether DKO neurons displayed deficits in GluA1 or GluA2 trafficking. To address this, we performed biotinylation assays to quantify the amount of surface and internal GluA1/GluA2 subunits in WT and DKO hippocampal cultures. As expected, DHPG application induced significant endocytosis of GluA1 subunits in WT cells, which was blocked by pre-incubation with SB (Fig. 4a, Supplementary Fig. 1) showing that p38 regulates mGluR-dependent AMPAR endocytosis. However, no change in the amount of GluA1 at the cell surface or accumulated inside of the cell was observed in DKO cells regardless of treatment (Fig. 4b, Supplementary Fig. 1). Surprisingly, no significant change in the amount of GluA2 at the cell surface or inside the cells was seen regardless of treatment or genotype (Fig. 4c,d). AMPAR subunit composition at the postsynaptic density determines the functional properties of excitatory synapses in the brain. The majority of AMPAR in the postnatal brain contain the GluA2 subunit, which is impermeable to divalent cations, including Ca^2+^, and displays a linear current–voltage (*I*/*V*) relationship^25^. In adult hippocampal pyramidal neurons, most of AMPAR are composed of GluA1 and GluA2 heterodimers and the proportion of GluA2-lacking AMPARs at synapse surface can increase in response to changes in synaptic activity^25^. In contrast to GluA2-containing receptors, GluA2-lacking AMPARs exhibit strong inward rectification, faster deactivation kinetics and higher conductance, contributing to larger, rapidly decaying synaptic events^25^,^26^. Thus, our findings from the biotinylation assays suggest that changes in the biophysical properties of mEPSC recorded in WT and DKO neurons result from differences in AMPAR subunit composition. To test this hypothesis, we measured the rectification index and kinetics of mEPSC under basal conditions and 30 min after DHPG exposure. Under basal conditions, we observed an increased rectification index in DKO cells compared with WT (Fig. 4e) suggesting an increased number of GluA2-containing AMPAR at synapses. Furthermore, DHPG exposure caused an increased rectification index in WT, but not DKO cells, an effect most likely caused by increased endocytosis of GluA2-lacking AMPAR. This observation is in agreement with the biotinylation data showing a significant reduction of GluA1, but not GluA2 at the cell surface after DHPG exposure in WT, but not in DKO cells (Fig. 4e).

Consistent with our hypothesis that DKO cells have reduced levels of GluA1 subunits at the synapses, the decay time constant of mEPSC was increased in DKO cells under basal conditions compared with WT (Fig. 4e). Increased decay time is most likely related to increased incorporation of GluA2 subunits at synapses, slowing deactivation and desensitization of the AMPAR^25^,^26^. Although no significant changes in total GluA1/2 protein expression were seen in cultures from both genotypes, it is important to point out that there is a ~20% reduction in expression of total GluA1, and the inverse in GluA2, in DKO cultures compared with WT (Fig. 4f,g).

Considering that DKO neurons displayed abnormal spine morphology and had deficits in glutamatergic synaptic transmission, it is tempting to suggest that the p38-MK2/3 cascade is required for actin remodelling and that absence of MK2/3 promotes the formation of inflexible and thinner spines, which express a reduced amount of GluA1 subunits at the cell surface. In WT cells, there is a ~60% reduction in the amount of GluA1 at the surface after DHPG exposure, whereas in DKO cells, there is no significant change in the amount of GluA1 at the surface (Fig. 4). Remarkably, DHPG failed to induce LTD in both WT cells pre-treated with SB and DKO cells, clearly demonstrating that the p38-MK2/3 cascade is required for GluA1 endocytosis, which underlies mGluR-LTD.

### The p38-MK2/3-cofilin cascade is required for DHPG-LTD

**Figure 5:**
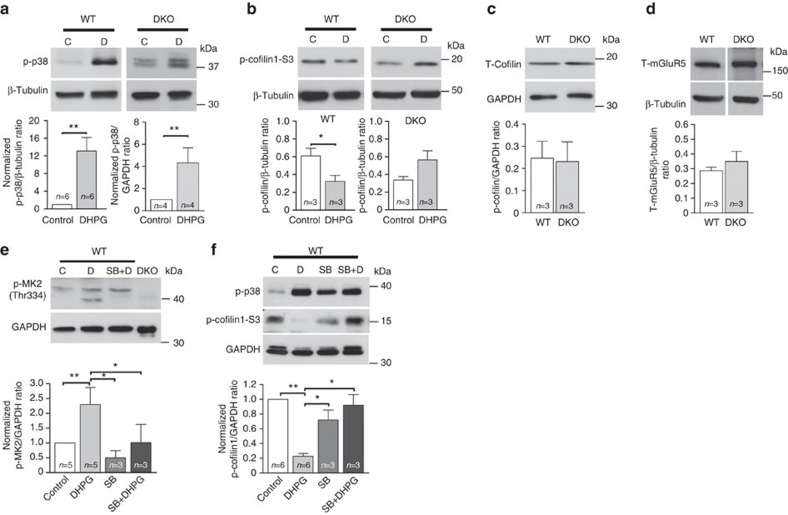
The p38-MK2/3-cofilin1 cascade is required in DHPG-LTD. (**a**–**f**) Representative blots of non-stimulated (C), DHPG-incubated (100 μM, 10 min; D), pre-incubated with p38 inhibitor SB 203580 (5 μM) alone (SB) or pre-incubated with DHPG application (SB+D) cultures from WT and MK2/3 DKO hippocampus. (**a**) DHPG causes a significant increase in p-p38 at (Thr180/Tyr182) and (**b**) a significant reduction in p-cofilin1 in WT cells, an effect that is blocked in DKO cells. Note that no change in total cofilin1 (**c**) or mGluR5 (**d**) expression is detected between genotypes. (**e**) Blot showing a significant increase in phosphorylation of MK2 (Thr334) after DHPG-exposure in WT cells, an effect blocked by pre-incubation with SB. (**f**) Blot showing that the DHPG-dependent reduction in levels of p-cofilin1 (Ser3) is blocked by pre-incubation of p38 inhibitor SB 203580 (5 μM) in WT hippocampal cultures. Incubation of SB alone does not promote any significant changes in cofilin1 phosphorylation. Glyceraldehyde 3-phosphate dehydrogenase (GAPDH) and β-tubulin were used as loading controls. Western blot band densitometry analyses were obtained from a minimum of three different primary hippocampal preparations from the WT and DKO mice. *T*-tests, one-way analysis of variance and the appropriate *post-hoc* test were conducted accordingly for each data set. Error bars indicate ±s.e.m. and *P<0.05, **P<0.01.

**Figure 6:**
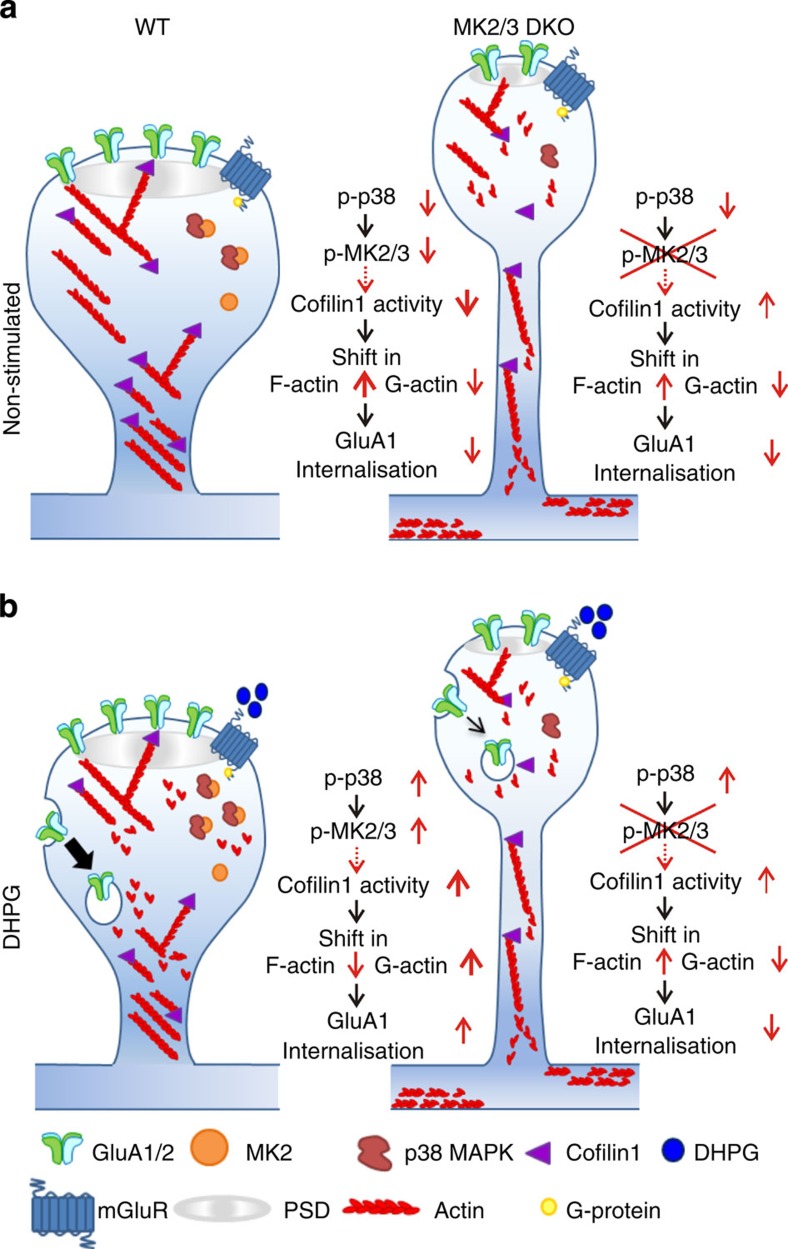
The p38-MK2/3 cascade regulating actin rearrangement in DHPG-LTD. (**a**,**b**) A schematic representation of the putative mechanism by which the p38-MK2/3 cascade regulates cofilin1 activity and GluA1 trafficking. (**a**) At non-stimulated dendritic spines of WT neurons levels of p38, MK2 and cofilin1 activity are low, and the balance between G- and F-actin is skewed towards F-actin; resulting in low levels of GluA1/2 endocytosis. By comparison, in spines of non-stimulated DKO neurons, where the p38-MK2 signalling cascade is blocked, there is a long lasting increase in basal cofilin1 activity levels, resulting in increased G-actin and abnormal spine morphology. Interestingly, the spines of MK2/3 DKO neurons, which display decreased head diameters and longer neck lengths, also have deficits in glutamatergic synaptic transmission. (**b**) Activation of mGluRs in WT spines induces an increase in p38 and MK2 activity, which correlates to an increase in cofilin1 activity and a shift in balance between G- and F-actin towards G-actin and increased endocytosis of GluA1 receptors. In DKO mice, the block of p38-MK2/3 cascade results in reduced cofilin1 activity and GluA1 endocytosis combined with deficits in mGluR-LTD.

Cofilin1 directly regulates actin polymerization at spines during synaptic plasticity^14^. Furthermore, cofilin1 activity, which is negatively regulated by phosphorylation at serine 3, has been shown to be regulated by MK2 (refs 11, 13, 16). We considered whether the activation of p38-MK2/3-cofilin1 cascade is required for mGluR-LTD. Hippocampal cultures from WT and DKO mice were treated with DHPG and the levels of p38, MK2 and cofilin1 activation determined. A significant increase in p38 phosphorylation at the activating sites was observed in DHPG-treated WT and DKO cells compared with control, showing that mGluR agonist binding can activate p38 in the absence of MK2/3 kinases (Fig. 5a, Supplementary Fig. 2). Interestingly, following DHPG-dependent activation of p38, a significant decrease in cofilin1 phosphorylation (p-cofilin1) at Ser3 is observed in WT cells, compared with control. In DKO cells, however, the levels of p-cofilin1 under basal conditions were similar to those in WT cells exposed to DHPG; and DHPG exposure caused only a slight increase in p-cofilin1 level (Fig. 5b). As no difference in total mGluR5 and cofilin1 expression was seen between genotypes (Fig. 5c,d), these observations suggest that in WT cells, the DHPG-dependent increase in cofilin1 activity shifts the ratio between F- and G-actin towards G-actin, which induces spine shrinkage and promotes GluA1 endocytosis. However, in DKO cells, DHPG exposure does not induce changes in cofilin1 activity and fails to promote the shift towards G-actin. Concomitantly, in WT cells, we also observed a DHPG-dependent increase in phosphorylation at threonine 334, a MK2 site phosphorylated by p38, and a decrease in cofilin1 phosphorylation at Ser3 (Fig. 5e,f, Supplementary Fig. 2). Pre-incubation with SB blocked the DHPG-induced cofilin1 dephosphorylation at Ser3 (Figs 3f and 5f), whereas no changes in p-cofilin1 levels were observed with SB alone, SB+DHPG and under control conditions (Fig. 5f). Taken together, these data suggest that the p38-MK2/3 cascade regulates DHPG-dependent actin remodelling through cofilin1 in hippocampal neurons (Fig. 6).

### MK2/3 DKO mice have deficits in mGluR-LTD

**Figure 7:**
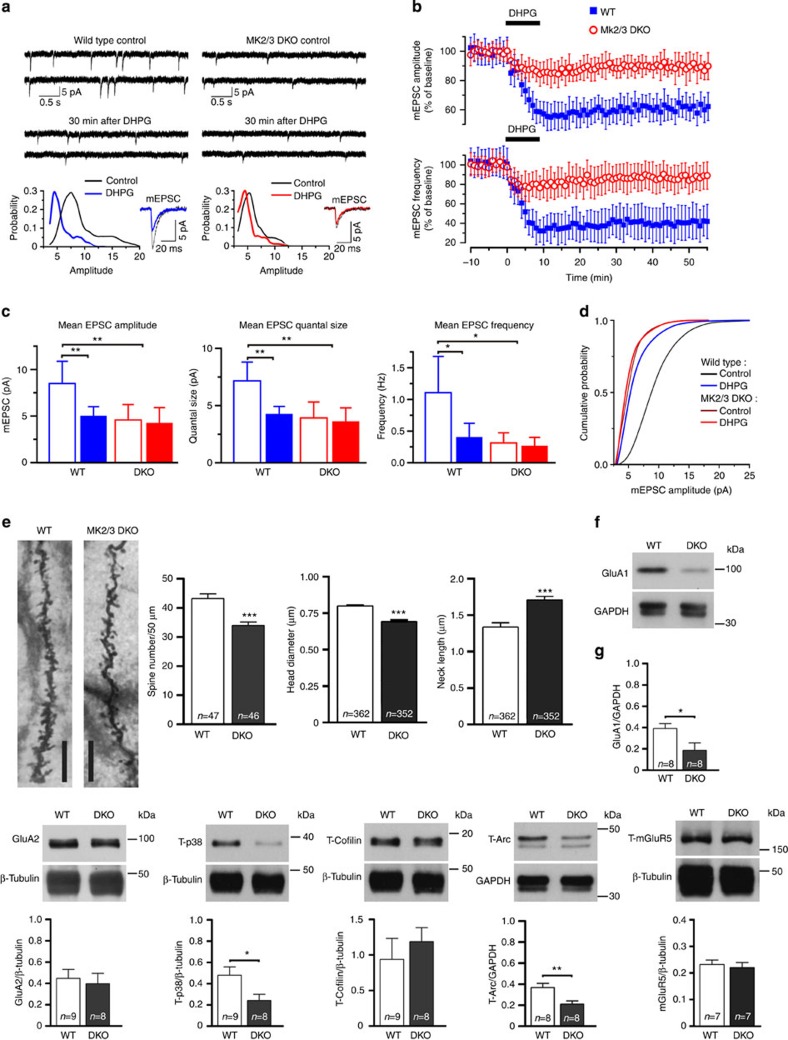
MK2/3 DKO mice have deficits in mGluR-LTD. (**a**) Examples of mEPSC traces from WT (left) and DKO neurons at baseline and 30 min after DHPG exposure. Bottom graphs show amplitude distributions recorded from the respective WT and DKO cells. Note that the unitary quantal size, indicated by the position of the main peak in the amplitude, is reduced after DHPG (100 μM) application suggesting a postsynaptic effect in WT cells, but no changes in quantal size is observed in DKO cells. (**b**) Average time course of changes in mEPSC amplitude (top graph) and frequency (bottom graph) induced by DHPG exposure. Mean LTD amplitude of WT CA1 pyramidal neurons was 62.6±9.6% of baseline at 45±5 min after DHPG exposure (blue line; *n*=9 cells from five animals) compared with 88.4±4.6% in DKO cells (red line; *n*=8 cells from five animals), and the mean mEPSC frequency was 41.7±21.9% of the baseline in WT compared with 81.3±16.2% in DKO cells. (**c**) Mean mEPSC amplitude (top), quantal size (middle) and frequency (bottom) across the two genotypes. Error bars=±s.e.m. and *P<0.02, **P<0.005. (**d**) Cumulative distributions for mEPSCs recorded in the CA1 pyramidal hippocampal neuron of WT (*n*=9 cells) and DKO (*n*=8 cells) from five independent WT and DKO animals. (**e**) Golgi impregnated images of processes from CA1 pyramidal cells. A significant decrease in spine density per 50 μm (WT: 43.2±1.6 spines, 46 cells; DKO: 33.9±1.2 spines, 47 cells), head diameter (WT: 0.8 μm±0.007; DKO: 0.7 μm±0.005) and an increase in neck length (WT: 1.34 μm±0.07; DKO: 1.71 μm±0.05) is observed in DKO mice compared with WT. Data were obtained from three different WT and DKO mice. Scale bar, 10 μm. (**f**,**g**) Blots showing protein levels for GluA1 and GluA2, p38, cofilin1, Arc/Arg3.1 and mGluR5 obtained from lysate of WT and DKO adult hippocampus. (**e**) There is a significant reduction in total p38, Arc/Arg3.1 and GluA1 proteins in DKO compared with WT, but not for GluA2, mGluR5 and cofilin1. Glyceraldehyde 3-phosphate dehydrogenase (GAPDH) and β-tubulin were used as loading controls. An unpaired *t*-test was performed. Error bars=±s.e.m. and *P<0.05, **P<0.01, ***P<0.001.

To further investigate the role of the p38-MK2/3 cascade in DHPG-LTD, we recorded mEPSC from CA1 pyramidal neurons in WT and DKO acute slices (4- to 5-week-old mice), as they replicate many aspects of the *in vivo* context. In agreement with hippocampal cultures, DHPG exposure (100 μM, 10 min) induced a significant reduction in amplitude and frequency of mEPSC in hippocampal pyramidal neurons from WT slices (Fig. 7a–c). No significant changes in mEPSC amplitude and frequency were observed in DKO pyramidal cells after DHPG application. In fact, mEPSC amplitude and frequency in DKO neurons under both basal conditions and following DHPG application were similar to those values observed after DHPG application in WT cells (Fig. 7a–c). Mean quantal size and cumulative probability (Fig. 7d) confirmed that DHPG application did not induce any change in AMPAR activity in CA1 pyramidal neurons of DKO cells. To test whether reductions in mEPSC amplitude and frequency observed in the CA1 hippocampal region were associated with morphological changes in dendritic spines, we analysed Golgi impregnated fixed brain sections from WT and DKO mice. Analysis of spines from CA1 hippocampal pyramidal neurons revealed significant reductions in spine density and head diameter, but increased spine neck length in DKO compared with WT mice (Fig. 7e). We then used hippocampal lysate from WT and DKO mice to determine expression levels of GluA1, GluA2, p38, cofilin1, mGluR5 and Arc/Arg3.1. Rapid increases in Arc/Arg3.1 protein expression is required to promote AMPAR endocytosis during DHPG-LTD^22^. Interestingly, a significant decrease in total GluA1, but not GluA2, mGluR5 or cofilin1 was seen in DKO compared with WT lysate (Fig. 7f,g, Supplementary Fig. 3), supporting our hypothesis that the deficits in mGluR-LTD seen in DKO cells is promoted by deficits occurring downstream of p38-MK2/3. Further corroborating this hypothesis, a significant reduction in Arc/Arg3.1 expression was observed in DKO hippocampal lysate. A reduction in p38 α/β expression was also observed in DKO hippocampal tissue. This observation was expected as p38-MK2/3 forms stable complexes^12^, and in the absence of MK2/3, p38 α/β is unstable and easily targeted for degradation. Collectively, these data show that, under our experimental conditions, exposure to DHPG induces a significant decrease in mEPSC amplitude and quantal size in WT CA1 pyramidal neurons, which is impaired in DKO CA1 neurons. Supporting the assumption that impaired DHPG-LTD observed in DKO neurons is likely due to a postsynaptic effect, we observed that DKO CA1 hippocampal neurons displayed longer and thinner spines together with a reduction in expression of total GluA1 and Arc/Arg3.1 protein in the hippocampus (Fig. 7f,g), proteins closely associated with mGluR-LTD^5^,^22^.

**Figure 8:**
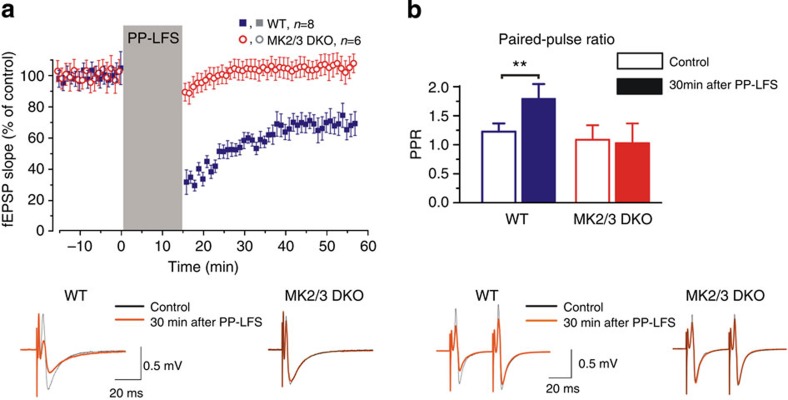
PP-LFS failed to induce mGluR-LTD in MK2/3 DKO mice. (**a**) Application of PP-LFS (900 paired-pulses delivered at 1 Hz, with an inter-pulse interval of 50 ms) induced LTD of the field excitatory postsynaptic potential (fEPSP) in the CA1 region of WT hippocampus (blue square) but failed to induce LTD in DKO (red circle). Dots in the graphs represent the average slope for six consecutive fEPSPs; data are shown as mean±s.d. for number of experiments indicated. Averaged fEPSP (20 consecutive responses) obtained from one individual WT and DKO slices at baseline (black line) and 30 min post PP-LFS application (red line). (**b**) Pooled data showing the significant increase in paired-pulse ratio following PP-LFS in WT mice (control: 1.21±0.14; after LTD: 1.78±0.25), an effect that is absent in DKO mice (control: 1.07±0.24; after LTD: 1.01±0.33). Averaged fEPSP (20 consecutive events) obtained from one individual WT and DKO slices at baseline (black line) and 30 min post PP-LFS application (red line). Error bars indicate±s.d. and **P<0.01.

To further characterize the involvement of MK2/3 cascade regulating mGluR-LTD, we used a paired-pulse, low-frequency stimulus (PP-LFS; 900 paired stimuli at 1 Hz with 50 ms inter-pulse interval) to synaptically induce mGluR-LTD^15^. In agreement with previous studies, delivery of PP-LFS at the Schaffer collateral-commissural pathway resulted in sustained depression of synaptic transmission in the CA1 hippocampal region of WT mice, an effect not observed in DKO mice (Fig. 8a). To elucidate whether the deficits in mGluR-LTD seen in DKO hippocampus resulted from presynaptic changes in neurotransmitter release, we compared the paired-pulse ratio (PPR) at baseline and 30 min after PP-LFS. Application of PP-LFS promoted an increase in the PPR in WT, but not in DKO mice (Fig. 8b). Increase in the PPR during mGluR-LTD, which is classically interpreted as an increase in release probability, has previously been shown to result from enhancement of neurotransmitter release, related to the activity of postsynaptic protein phosphatases^23^. Combined, our findings clearly demonstrate that the MK2/3 cascade regulates chemically (DHPG) and synaptically induced mGluR-LTD, most likely by postsynaptic mechanisms.

### MK2/3 DKO mice have deficits in spatial reversal learning

Previous studies have shown that mGluR5 knockout mice, which have impaired mGluR-LTD in the hippocampal CA1 region, have only mild deficits in acquisition/consolidation of spatial learning, but poor performance on task reversal^27^,^28^. Furthermore, considering that: (i) mGluRs play a critical role in acquisition of new memories and reversal learning^27^,^28^; (ii) DHPG-induced LTD requires activation of mGluR5 (ref. 29) and (iii) in DKO mice mGluR-LTD is impaired, we investigated whether DKO mice had deficits in hippocampal-dependent spatial learning. We used a modified Barnes maze task^30^, independent of motivating factors (for example, aversive stimuli)^31^ and requiring the use of external reference landmarks to locate the exit hole. Animals were tested for 20 consecutive days, grouped into 5 day blocks (blocks 1–3 representing acquisition, consolidation and expression phases of learning). On day 16 (and all block 4), the platform was rotated 180°, requiring animals to learn a new location for the exit hole. No adjustments were made for variability in learning ability.

**Figure 9:**
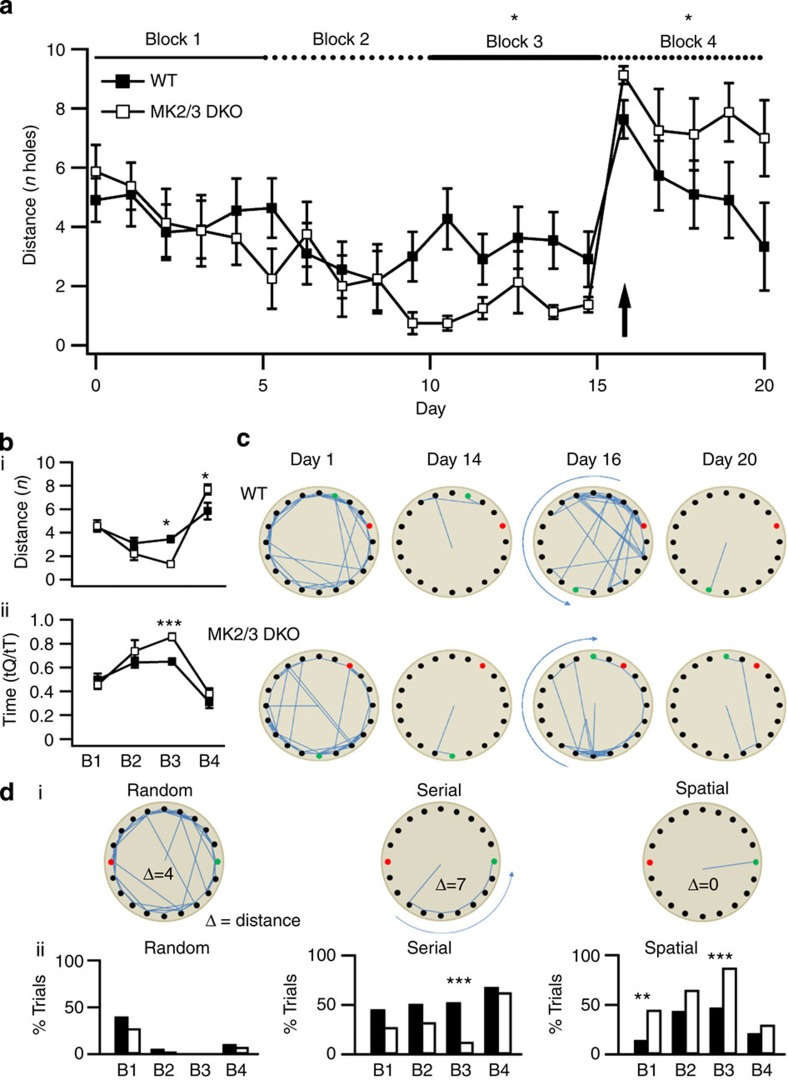
MK2/3 DKO mice have deficits in reversal learning. (**a**) Daily average distance from the first hole visited and exit hole location (distance) for WT (*n*=11) and DKO (*n*=8) animals (F(7.364)=18.344; *P*<0.001, analysis of variance). By block 3, DKO animals showed a significant difference in spatial orientation compared with WT (mean distance from exit was 1.33±0.24 holes in DKO, 3.46±0.42 holes in WT). Following rotation of the exit hole by 180°, WT acquired the new orientation faster than DKO, who consistently returned to the original quadrant over block 4 (mean distance was 5.68±0.54 in WT; 7.68±0.49 in DKO). (**b**) Block averages for (i) distance and (ii) time spend in quadrant for WT and DKO. DKO animals showed a significant increase in time spent in the quadrant in block 3 (ratio of time was 0.46±0.04 in block 1, 0.86±0.03 in block 3, *T*-test). (**c**) Maps plotting hole visitation of an individual WT and DKO animal on day 1 (novelty), day 14 (task embedded), day 16 (180° rotation) and day 20 (final day). (**d**(i)) Maps showing example search strategies utilized by the animals and distance calculations (Δ), (**d**(ii)) Pooled data showing proportions of animals displaying random, serial or spatial search strategies in each block. DKO animals used significantly more spatial search strategies than WT in blocks 1 and 3 (45% of trials compared with 16% in WT in block 1, *P*=0.006 when compared with serial; 88% of trials when compared with 47% in WT in block 3, *P*<0.001 when compared with serial, Fisher’s-exact test), correlated to a significant drop in serial searches in block 3. Both groups used serial strategies on rotation of the exit hole. Error bars indicate ±s.e.m. and *P<0.05, **P<0.01, ***P<0.001.

Spatial learning was indicated by the distance between the first hole visited and the exit hole location. No differences were observed in blocks 1 and 2, suggesting similar learning profiles for the task (Fig. 9a). By block 3, however, DKO animals exhibited stronger directionality towards the exit hole than WT (Fig. 9a,b). In WT, distance fluctuated around random, with mice successfully locating the exit independent of first hole position, which suggests increased spatial flexibility. On 180° rotation of the exit (block 4), DKO animals showed significant deficits in ability to adjust their learning. WT animals rapidly adjusted their orientation over block 4, whereas DKO animals failed to show improvement over the block (Fig. 9a,b).

Dynamic components of learning were indicated by search strategy (Fig. 9c,d). Significant differences in search strategy were observed between WT and DKO mice. On day 1, WT animals mainly used random strategies (multiple maze crossings to other quadrants), whereas DKO used random or serial (sequential hole-to-hole) approaches. Over block 1, DKO animals preferentially applied serial strategies, whereas WT used random or serial strategies. By block 3, with the task embedded and the mice oriented to the exit location, DKO mice used mainly a spatial strategy (direct to the exit ±2 holes), whereas WT mice used either serial or spatial strategies. On 180° rotation of the exit, both WT and DKO used serial strategies, the most efficient for finding target locations. However, DKO mice failed to re-learn the new location of the exit hole. These findings suggest that MK2/3 is a key regulator of mGluR-LTD and hippocampal-dependent spatial reversal learning.

## Discussion

The present study identifies MK2/3 as a link between p38-dependent mGluRs activation and the endocytosis of GluA1 subunits underlying mGluR-LTD^1^,^3^,^22^. Here we show that the MK2/3 cascade regulates dendritic spine morphology and is required for mGluR-LTD in hippocampal neurons (Fig. 6). Compared with WT, the spines of DKO neurons have a strikingly long neck and reduced head diameter, morphological characteristics that parallel the effect of the mGluR-agonist DHPG on hippocampal dendritic spines^5^,^6^. Interestingly, re-insertion of MK2-WT, but not MK3-WT, into DKO neurons reversed the deficits in spine morphology, and restored basal synaptic transmission to WT levels. Furthermore, MK2 activation was sufficient to induce changes in actin cytoskeleton at spines, as overexpression of a construct mimicking activated MK2 in DKO cells rescued spine neck length and head diameter, whereas overexpression of a MK2 kinase-dead mutant in WT cells produced the DKO phenotype. Somewhat unexpectedly, expression of the kinase-dead MK2 protein in DKO cells also caused an increase in spine neck length. This may result from the kinase-dead MK2 still being able to interact with its downstream effectors (such as LIM kinase1, LIMK1). As the inactive MK2 is unable to phosphorylate these effector proteins, it may instead sequester them, reducing their activity even further (that is, below their baseline activity).

Supporting our hypothesis that the MK2/3 cascade is required for mGluR-LTD, the mGluR-dependent reduction in mEPSC amplitude and frequency seen in WT cells is impaired in DKO cells. One explanation for this observation is that the absence of MK2/3 results in spines with reduced head diameter, which accommodate less AMPAR at the surface and DHPG exposure is unable to activate the mGluR-p38-MK2/3 signalling cascade required to promote rapid actin-remodelling and GluA1 endocytosis underlying LTD. These findings strongly suggest that loss of MK2/3 activity is responsible for the spine abnormalities and deficits in glutamatergic synaptic transmission observed in DKO cells.

Further evidence that the p38-MK2/3 cascade is responsible for deficits in DKO mEPSC amplitude and quantal size after DHPG exposure, is that pre-incubation of WT cells with a p38 inhibitor blocks the reduction in these parameters. Similarly, re-insertion of MK2-WT, but not MK3-WT, into DKO cells before DHPG application rescued mEPSC amplitudes. In agreement with mEPSC recordings, p38 inhibitors blocked the DHPG-dependent intracellular accumulation of GluA1 observed in WT cells. Taken together, these findings suggest that decreased mEPSC amplitude and quantal size observed under basal conditions and after DHPG exposure in DKO neurons results from changes at the postsynaptic membrane. However, a contributing presynaptic effect cannot be excluded; and further evidence is required to determine whether vesicle release is altered in DKO mice.

Supporting our observations in cultured hippocampal neurons, application of DHPG in WT hippocampal CA1 pyramidal neurons promoted a significant decrease in AMPAR-mediated mEPSC amplitude, quantal size and frequency, an effect that was impaired in DKO. Similar to findings in cultured neurons, the properties of DKO mESPC had similar values to those seen in WT cells exposed to DHPG. Comparable to the abnormal spine morphology seen in DKO hippocampal cultured neurons, a significant decrease in spine density, head diameter and increased spine neck length was observed in Golgi-stained CA1 pyramidal neurons in DKO tissue compared with WT. Another important observation that may explain the reduced AMPAR-dependent transmission under basal conditions observed in DKO mice was the significant reduction in total GluA1 and Arc/Arg3.1 protein expression compared with WT. Interestingly, no significant changes were observed in total mGluR5, GluA2 and cofilin1 proteins. Previous studies have associated hippocampal mGluR-LTD with decreases in either frequency^6^,^22^,^23^ or both frequency and amplitude of mEPSC^32^. Interestingly, the majority of these studies suggest that the molecular machinery crucial for induction and expression of mGluR-LTD is located postsynaptically, including actin remodelling, GluA1 endocytosis, protein tyrosine phosphatases and local Arc/Arg3.1 translation^6^,^22^,^23^. Here we provide strong evidence to show that p38-MK2/3-dependent mGluR-LTD is regulated by postsynaptic GluA1 endocytosis, resulting in decreased mEPSC amplitude and quantal size. In agreement with previous studies, we also observed a decrease in mEPSC frequency, which can be explained either by changes in presynaptic neurotransmitter release or by postsynaptic mechanisms in which complete AMPAR endocytosis occurs, silencing the postsynaptic density^33^.

The requirement of cofilin1 activity in regulating actin dynamics and its role in synaptic plasticity is well documented^4^,^14^. However, the signalling cascade(s) controlling cofilin1 activity in mGluR-LTD are far from resolved. Here we show that significant increases in DHPG-dependent p38 and MK2 activity correlates with decreased phosphorylation of cofilin1 at Ser3 in WT hippocampal cells, suggesting that the p38-MK2-cofilin1 cascade is required for actin-remodelling occurring in mGluR-LTD. As expected, pre-incubation with SB blocked the DHPG-dependent decrease in cofilin1 Ser3 phosphorylation. Interestingly, no change in DHPG-dependent cofilin1 Ser3 phosphorylation level, and therefore activity, was observed in DKO cells. Furthermore, levels of phosphorylated cofilin1 in DKO cells under basal conditions were reduced compared with WT. As no change in total cofilin1 expression was seen between genotypes, we suggest that MK2/3 activity is required to promote the shifts in cofilin1 activity required to induce mGluR-LTD^6^, a mechanism impaired in DKO cells. The missing piece of the puzzle in this cascade is the link between mGluR-dependent activation of the p38-MK2/3 cascade and the decrease in cofilin1 phosphorylation observed in mGluR-LTD. The integration between LIMK1, slingshot 1 phosphatase (SSH1) and actin may provide some clues. One possible mechanism is that in WT neurons under basal conditions, LIMK1 is activated at threonine 508 independently of mGluR-p38-MK2 (MK2 phosphorylates and activates LIMK1 at Ser323), maintaining the high levels of cofilin1 phosphorylation^6^,^13^,^34^. Following mGluR-activation, increasing p-p38 and p-MK2 further increases LIMK1 activation by phosphorylating Ser323, a fact that contrasts with decreased cofilin1 phosphorylation observed in our findings. Additional increases in LIMK1 phosphorylation caused by mGluR-p38-MK2 activation may result in further and immediate increases or stabilization of F-actin at the spines, which consequently activates SSH1. Once activated, SSH1 simultaneously dephosphorylates LIMK1 and cofilin1 (ref. 34). However, we cannot exclude the possibility that an unknown p38-MK2/3 downstream target, independent of LIM kinases, is required for regulating mGluR-dependent cofilin1 activity. In fact, a recent study has shown that activation of bone morphogenetic protein-2, a member of the transforming growth factors, requires the p38-MK2-small heat-shock protein 25 cascade to promote actin remodelling during C2C12 migration^16^. Understanding the signalling cascade controlling cofilin1 is vital, as increasing evidence implicates cofilin1-mediated actin dynamics in synaptic plasticity and memory formation^35^.

Previous studies have shown that mGluRs are implicated in reversal learning and memory formation^27^,^28^. Here we show that DKO mice, which have deficits in hippocampal mGluR-LTD, retain the capacity to acquire a hippocampal-dependent spatial learning task but are unable to exhibit reversal learning. In comparison to a number of previous studies^36^, the Barnes maze task used here did not use motivating cues to aid learning, making it a more naturalistic process dependent on individual drive to complete the task. Interestingly, no differences were observed between genotypes during the first two blocks, where the animals learnt and embedded the task. However, by block 3, DKO mice performed more efficiently than WT littermates, suggesting a stronger focus on task completion. After rotating the exit hole by 180°, DKO mice persisted in returning to the original location, suggesting an inability to overwrite the learnt task with new information. Fixation on tasks and inability to switch are reminiscent of the characteristic behavioural inflexibility seen in autism and schizophrenia^37^,^38^. Although mechanisms underlying cognitive flexibility are not fully established and much debated, our data show unequivocally that the absence of MK2 and 3 impacts on behavioural and cognitive function.

The results presented here identify, for the first time, a requirement for the MK2/3 cascade in neuronal function. Our data show that MK2/3 regulates synapse transmission, most likely at the postsynaptic site, is required for mGluR-dependent LTD and plays a critical role in hippocampal-dependent cognitive flexibility. These findings provide the crucial mechanistic link between mGluR-dependent activation of p38 and the trafficking of postsynaptic AMPAR regulating mGluR-LTD and may provide novel therapeutic targets to slow the cognitive decline associated with natural ageing and neurodegenerative disorders.

## Methods

### Generation of MK2/3 DKO mice

All animals used in this study were treated in accordance with UK Animal (Scientific Procedures) legislation and under the appropriate project licenses, national and local ethical approval. Sample size for slice experiments were calculated using variance from previous experiments to indicate power, with statistical significance set at 95%. Replication values are incorporated in the figures, where appropriate.

Mice heterozygous for both MK2 and MK3 isoforms were used for breeding to generate MK2/3 WT and MK2/3^−/−^ DKO mice strains. The MK2^−/−^ mouse was first generated by introducing a neomycin-resistance gene into the exon encoding subdomains V and VI within the catalytic domain of MK2. Consequently, stop codons were introduced into all reading frames thus producing a truncated form of MK2 lacking amino acids 130–383, which are essential for MK2 catalytic activity. This mutation was carried out in embryonic stem cells by homologous recombination and these cells were injected into C57BL/6J blastocysts to generate MK2 KO mice^39^. A MK3 KO was generated via Cre-mediated deletion as previously described^40^. Heterozygotes were mated in-house to generate the DKO mice. The MK2/3 DKO mice are viable and fertile, however, breeding rate is considerably low. No obvious behavioural or brain defects were observed between littermates. Animals used for slice preparation, protein extraction or Golgi staining were killed by cervical dislocation. To prepare lysate from the hippocampi, brains from WT and MK2/3 DKO mice were quickly removed and placed in ice-cold artificial colony-stimulating factor consisting of (mM): 124 NaCl, 3 KCl, 26 NaHCO_3_, 1.25 NaH_2_PO_4_, 2 CaCl_2_, 1 MgSO_4_ and 10 D-glucose (bubbled with 95% O_2_–5% CO_2_), and hippocampi extracted from the brain using a dissecting microscope (Leica, LED 1000). Hippocampal slices were obtained from male and female C57BL/6 WT and MK2/3 DKO mice aged 4–5 weeks.

### Constructs

Constructs used were: actin-eGFP (pAcGFP1-Actin, Clontech) and pEGFP (PT3051-5, Clontech). The pEGFP-C1-MK2-WT, pEGFP-C1-MK3-WT, pEGFP-C1-MK2-EE and pEGFP-C1-MK2-K79R constructs were generated as described previously^17^,^39^,^40^.

### Hippocampal cell culture

Mouse hippocampal neuronal cultures were prepared from postnatal day 0 or 1 mice as described previously^41^,^42^. Briefly, hippocampi were extracted from the brain at 4 °C, and subject to digestion with trypsin (Sigma-Aldrich) and mechanically dissociated with DNAse (Sigma-Aldrich). Cells were plated onto either 22-mm glass coverslips in 35 mm Petri dishes or directly into 60 mm Petri dishes coated with poly-L-lysine hydrobromide (0.5 mg ml^−1^, Sigma-Aldrich). The plating medium consisted of Neurobasal-A medium (Gibco) supplemented with gentamycin (ForMedium), L-glutamine (ForMedium), 2% B27 (Gibco) and 5% horse serum (Gibco). The following day, the plating medium was changed for horse serum-free feeding medium. Cultures were maintained in an incubator environment of 95% O_2_, 5% CO_2_ and fed every 5–7 days. For all the hippocampal cultured experimental work, cells were used at 14–16 days *in vitro*. For immunocytochemistry, cells were transfected using Lipofectamine 2000 (Invitrogen). Neurons were used 15–16 h after transfection.

### DHPG stimulation

For DHPG-LTD experiments, hippocampal cultures from WT and MK2/3 DKO mice were washed with warm HEPES-buffered saline (HBS) consisting of (mM): 119 NaCl, 5 KCl, 2 CaCl_2_, 2 MgCl_2_, 25 HEPES, 33 glucose (pH 7.2). For DHPG and SB+DHPG conditions, cultures were then stimulated with 100 μM (RS)-3,5-DHPG (3,5-dihydroxyphenylglycine) in HBS for 10min (DHPG-induced LTD). HBS was used as vehicle for control conditions. The GABA_A_ receptor antagonist picrotoxin (50 μM; Sigma-Aldrich) and NMDAR antagonist L-689,560 (trans-2-carboxy-5,7-dichloro-4-phenylaminocarbonylamino-1,2,3,4-tetrahydroquinoline; 5 μM, Tocris) were used throughout. The p38 inhibitor, SB 203580 (5 μM; Calbiochem), was added for 50 min prior DHPG exposure (SB+DHPG condition).

### Transfection and immunocytochemistry

To visualize dendritic spines, hippocampal cultures from WT and MK2/3 DKO mice were transfected with an actin-eGFP-tagged construct using Lipofectamine 2000 (Invitrogen) and used 15–16 h after transfection. After DHPG stimulation, neurons were fixed using 4% paraformaldehyde (PFA, pH 7.4) in HBS for 10 min, quenched with 0.1 M glycine and mounted onto coverslips using Mowiol. For MK2 rescue experiments, hippocampal cultures from WT and MK2/3 DKO were transfected with constructs (listed previously) using the same procedure described.

### Morphological analysis

For morphological analysis, the experimenter was blinded to the cDNA constructs transfected into the tissue, and animal genotype. Immunofluorescence was observed using a × 63 oil-immersion lens on an inverted Leica SP5 confocal microscope (Leica Microsystems). Fluorophores were excited with 488 or 568 nm wavelengths and emission from one single plane was detected through 505–530 nm band-pass or 560 nm long-pass filters. For imaging acquisition, the physical parameters such as laser intensity, gain and magnification were set up for control conditions; the settings were saved and used throughout the same experiment. Z-stacks were obtained at 0.2 μm steps and processed using Leica Microsystem LAS software (Version 2.6.0, Leica). The image calibration and the quantification of pixel intensity were performed as previously described^20^,^42^. Briefly, the TIFF images were imported into ImageJ software ( http://imagej.nih.gov/ij/) and scaled according to the physical parameters used for the images acquisition. For morphological analysis, a random primary or secondary process was selected and a line was drawn along the selected process and electronically measured. Spines along this process were manually counted. The neck length was measured from the base of the spine on the process to the base of the spine head, which was kept consistent throughout the analysis. ImageJ software was used to manually select each of the visible and measurable spine necks with the line tool. The line drawn along the spine neck was measured electronically to give an accurate measurement. To measure spine volume and spine head diameter, TIFF images were exported as compressed Z-stacks using ImageJ, opened and calibrated in Neuronstudio software and processes were automatically selected and spines identified manually^43^. Co-localization of pre- and postsynaptic markers was evaluated using ImageJ software^44^. Briefly, correlation between green and red fluorescence was calculated as intensity correlation quotient (ICQ) based on the relative differences from the mean for each pixel (PDM); the pseudo-colour PDM images were generated as an output of ImageJ analysis routine. The theoretical limits for ICQ are ±1; random staining should show ICQ 0, positive values are characteristic for dependent staining.

### Western blotting

Cultured hippocampal neurons from both WT and DKO mice were either incubated with vehicle (control) or stimulated with DHPG and pre-incubated with SB 203580 before DHPG stimulation (100 μM) for 10 min. Cells were then lysed with lysis buffer (1 mM EDTA, 1 M Tris–HCl (pH 7.5), 1% Triton X-100, 1 mM sodium orthovanadate, 50 mM sodium fluoride, sodium pyrophosphate, 0.27 M sucrose, 20% NaN_3_ and protease inhibitor cocktail), and the lysate rotated for 1 h at 4 °C. Samples were centrifuged at 13,000 r.p.m. for 15 min and the supernatant collected and protein levels were determined (BCA protein assay kit, Thermo Scientific). The proteins were separated using a SDS–polyacrylamide gel electrophoresis system and transferred onto Hydrobond-ECL membrane (GE Healthcare). The blots were incubated overnight at 4 °C with primary antibodies including phospho (p)-p38 MAPK (Thr180/Tyr182; Cell Signaling, 4511), total p38 MAPK (Cell Signaling, 9212), p-MK2 (Thr222/Thr334; Cell Signaling, 3316/3007), p-cofilin (Ser3) (Cell Signaling, 3313), total cofilin1 (Cell Signaling, 3312), GluA1 (Millipore, AB1504), GluA2 (Millipore, MAB397), Arc/Arg3.1 (Synaptic Systems, 156–003), β-Tubulin (Abcam, AB6046) and GAPDH (Abcam, AB8245). The appropriate secondary antibodies (Cell Signaling, anti-Rabbit IgG HRP-linked antibody, 7074 and Cell Signaling, anti-Mouse IgG HRP-linked antibody, 7076) were incubated for 1 h at room temperature and blots developed using ECL reagents (Thermo Scientific, Supersignal West Femto). Blots were then developed, scanned and densitometry of band analysed using Fiji software as described previously^20^. Hippocampal lysate prepared from isolated hippocampi from WT and DKO mice (P28–30) was also used to determine total levels of GluA1, GluA2, cofilin1, Arc/Arg3.1 and p38 protein.

### Biotinylation assays

High-density hippocampal cultures from WT and DKO mice were stimulated as described previously and the amount of surface and internal GluA1 protein analysed using a biotinylation assay described in Corrêa *et al*.^45^ Following DHPG stimulation, cells were transferred on ice and incubated with 0.25 mg ml^−1^ of EZ-Link Sulfo-NHS-SS-Biotin (Thermo Scientific, 21331) in ice-cold HBS for 15 min at 4 °C. After incubation, cells were washed three times with 50 mM NH_4_Cl in HBS for 5 min each at 4 °C and once with HBS for 5 min at 4 °C. Cells were then lysed, rotated for 1 h at 4 °C and samples centrifuged at 800 r.p.m. for 2 min. The supernatants were removed and protein levels determined using a BCA assay kit (Thermo Scientific). A measure of 100 μg of protein making 250 μl of final volume was incubated with 20 μl of pre-washed streptavidin-agarose beads (Sigma-Aldrich, S1638) and rotated for 3 h at 4 °C. Samples were centrifuged at 800 r.p.m. for 30 s to precipitate the beads. The supernatant was removed and the beads washed three times with lysis buffer containing protease inhibitor cocktail (Roche). Proteins were eluted from the beads with 20 μl of 5 × loading buffer, and the total amount of the eluted protein from the beads (surface protein, B; 20 μl) plus 10 μg of the input (total protein, T), as well as 25 μl (10% of total volume) of the bead supernatant (Internal condition, I) were loaded into an 8% SDS–polyacrylamide gel electrophoresis gels and separated using electrophoresis system. Blots were incubated overnight at 4 °C with primary antibody rabbit anti-GluA1 (Millipore, AB1504) and incubated with appropriate secondary antibodies to detect the surface and internal levels of GluA1 subunits. β-Tubulin was used as the loading control. Western blot band densitometry was analysed as previously described^20^. Values obtained from the supernatant and bead samples were normalized to the input within each of the conditions separately.

### Whole-cell mEPSC recordings

For mEPSC recordings in cultures, the experimenter was blinded to the cDNA constructs transfected into the tissue, and animal genotype. Spontaneous mEPSC were recorded in 15–18 days *in vitro* cultured pyramidal hippocampal neurons or in hippocampal slices obtained from 4- to 5-week-old WT and MK2/3 DKO as described previously^20^,^37^. Acquisition and analysis of mEPSC were performed as described previously^20^,^24^,^46^. To measure AMPAR-dependent mEPSC, pyramidal neurons were voltage clamped at −80 mV throughout the experiment with patch pipettes (4 Ω) filled with intracellular solution (10 mM HEPES; 50 mM KCl; 55 mM K-gluconate; 10 mM NaCl; 2 mM MgATP and 0.1 mM EGTA at pH 7.35) at 29–31 °C as previously described^37^. 1 μM tetrodotoxin, 50 μM picrotoxin to block GABA_A_ receptors and the NMDAR antagonist L-689,560 (5 μM) were present throughout recordings to isolate AMPAR currents. Induction of mGluR-LTD was achieved by adding DHPG (100 μM) in the extracellular solution for 10 min. The series and the input resistances were 4–8 MΩ and 500–1,300 MΩ, respectively, and varied by less than 20% in cells accepted for analysis. Currents were monitored using an AxoPatch200B patch-clamp amplifier (Axon Instruments) filtered at 2 kHz and digitized at 4 kHz. Acquisition and analysis of mEPSC were performed as described previously^20^. Experiments were controlled by PCI-6229 data acquisition board (National Instruments, USA) and WinWCP software (Strathclyde Electrophysiology Software). mEPSCs were analysed offline using a self-designed software as previously described^20^,^24^. For the initial detection of spontaneous events, inward transmembrane currents of amplitude >2 s.d. of the baseline noise were selected. After that, every single spontaneous event was analysed within a 60-ms time window and its amplitude and kinetics were determined by fitting a model curve with single exponential rise and decay phases. Mean square error of fit amounted to 5–15% of peak amplitude. Whenever error of fit exceeded 25%, spontaneous currents were discarded from further analysis. The amplitude distributions of spontaneous and evoked currents were analysed with the aid of probability density functions and likelihood maximization techniques, as previously described^20^; all amplitude distributions shown were calculated as probability. Synaptically induced LTD was induced by stimulating the Schaffer collaterals with 900 paired-pulses (50 ms inter-pulse interval) delivered at 1 Hz (ref. 15).

### Golgi staining

Brains were removed from P30-P79 mice, washed briefly with cold PBS and treated according to the vendor instructions for the FD Rapid GolgistainTM Kit (FD NeuroTechnologies, Inc.). Following the initial impregnation process, 200 μm parasagittal slices of the hippocampus were prepared using a vibratome (Microm, HM650V). The slices were mounted onto gelatin-coated microscope slides and left overnight at room temperature. The following day, the slices were further processed using the FD Rapid GolgistainTM Kit and coverslip mounted with Mowiol. The slices were then visualized using a × 100 oil-immersion lens on a Zeiss Axioskop FS transmission light microscope and imaged using a Nikon DXM1200F digital camera. The images were captured from Golgi-impregnated pyramidal neurons of the CA1 region of the hippocampus. The TIFF files were imported into ImageJ and Z-stacks compressed. Images were then calibrated accordingly to the acquisition parameters and the length of the process measured electronically as described previously, and spine numbers from primary and secondary processes were measured for both genotypes. Morphological analysis was performed with the experimenter blinded to the genotype of the animals. Spines neck length and head diameter were measured using Neurolucida software (MicroBrightField) on a Nikon 80i microscope (Nikon) equipped with a × 100, 1.25 numerical aperture oil-immersion lenses.

### Behavioural testing

Behavioural experiments were performed with the experimenter blinded to the genotype of the animals. Animals were standard housed with littermates, provided with food and water *ad libitum* and maintained on a 12:12 (light–dark) cycle. WT (*n*=11) and DKO (*n*=8) animals aged 28–30 days at training commencement were utilized. Spatial learning was assessed using a modified circular Barnes maze measuring (1 m diameter, situated 1 m from the floor and contained 20 × 5 cm holes, 5 cm evenly spaced around the perimeter), which in this version displayed 20 holes evenly distributed around the edges of maze, instead of 40 holes used in Bach *et al.*^30^ The maze contained an ‘exit’ box positioned under one of the holes and a ‘fake’ box (incorporated to mimic light reflection from the exit box but with no depth) under another. Holes were randomly assigned on the first day and maintained in this position for 15 days for the ‘exit’ box before 180° shift (see below) and all 20 test days for the ‘fake’ box. The maze was positioned centrally within the lab, with surrounding equipment and architectural features kept in fixed positions, acting as spatial cues for learning. Animals were placed in the centre of the maze, released and allowed to explore the maze. The task was completed when the animal entered the exit box. All runs were recorded using a camera system attached to a computer for offline analysis. Animals were tested daily and changes in speed and accuracy of task performance measured. On days 1–5 [Block 1], the exit box contained flavoured treats as a reward for task completion. On days 6–20, treats were awarded in the home cage on completion to prevent cued orientation of the exit box location via olfactory stimulation. On days 16–20 [Block 4], the position of the exit box was rotated 180° to determine the spatial component of and coping ability for the task. The experimenter was blind to animal genotype, which was confirmed on task completion. To determine spatial orientation to the task, the distance from first to the exit hole was calculated, this was maximal at *n*=10 and chance was considered to be *n*=5. The time spent within the quadrant containing the exit hole was also determined (the quadrant was calculated as the region bounded by the midpoint of the maze, extending out to perimeters set at two holes either side of the exit hole). Mapping the progression of the animals around the maze allowed determination of the search strategy used. These were as follows: random: with no consistent pattern, >2 crossings of the open field; serial: predominantly a hole-by-hole progression to the exit hole with <2 crosses of the open field; spatial: direct to the exit hole ±2 holes and no deviation outside of the quadrant (Fig. 9d)^7^.

### Statistical analysis

Data were analysed using Prism (Version 5.04, GraphPad) and SPSS statistics 21 (IBM) software. Unpaired *t*-tests, Mann–Whitney *t*-tests, Kolmogorov–Smirnov test, one-way analysis of variance and the corresponding *post-hoc* tests (Turkey or Dunn’s) were performed as appropriate.

## Author contributions

S.A.L.C. conceived and directed the project, designed the experiments, performed the immunocytochemistry experiments, confocal imaging and wrote the manuscript. K.L.E. performed and analysed the immunocytochemistry and biochemical experiments; the Golgi experiments and performed the all spine morphology analysis. T.O. performed initial biochemical experiments. J.M. performed initial experiments, contributed to the experimental design and to the interpretation of the results. M.G. provided the MK2/3 DKO mice and several constructs used in this study. D.R.C. designed, performed and analysed the behavioural experiments. O.P., S.R.-N., and Y.P. designed, performed and analysed the electrophysiological recordings.

## Additional information

**How to cite this article**: Eales, K. L. *et al.* The MK2/3 cascade regulates AMPAR trafficking and cognitive flexibility. *Nat. Commun.* 5:4701 doi: 10.1038/ncomms5701 (2014).

## Supplementary Material

Supplementary Information Supplementary Figures 1-3
