# Supplementary material for: The MK2/3 cascade regulates AMPAR trafficking and cognitive flexibility
Source: Nat Commun. 2014 Aug 19;5:4701. doi: 10.1038/ncomms5701 (PMC4143933; doi:10.1038/ncomms5701)
Supplement: Supplementary Information — Supplementary Figures 1-3 [file ncomms5701-s1.pdf]

Supplementary figure 1: AMPAR trafficking is disrupted in MK2/3 DKO neurons (related to Figure 4).

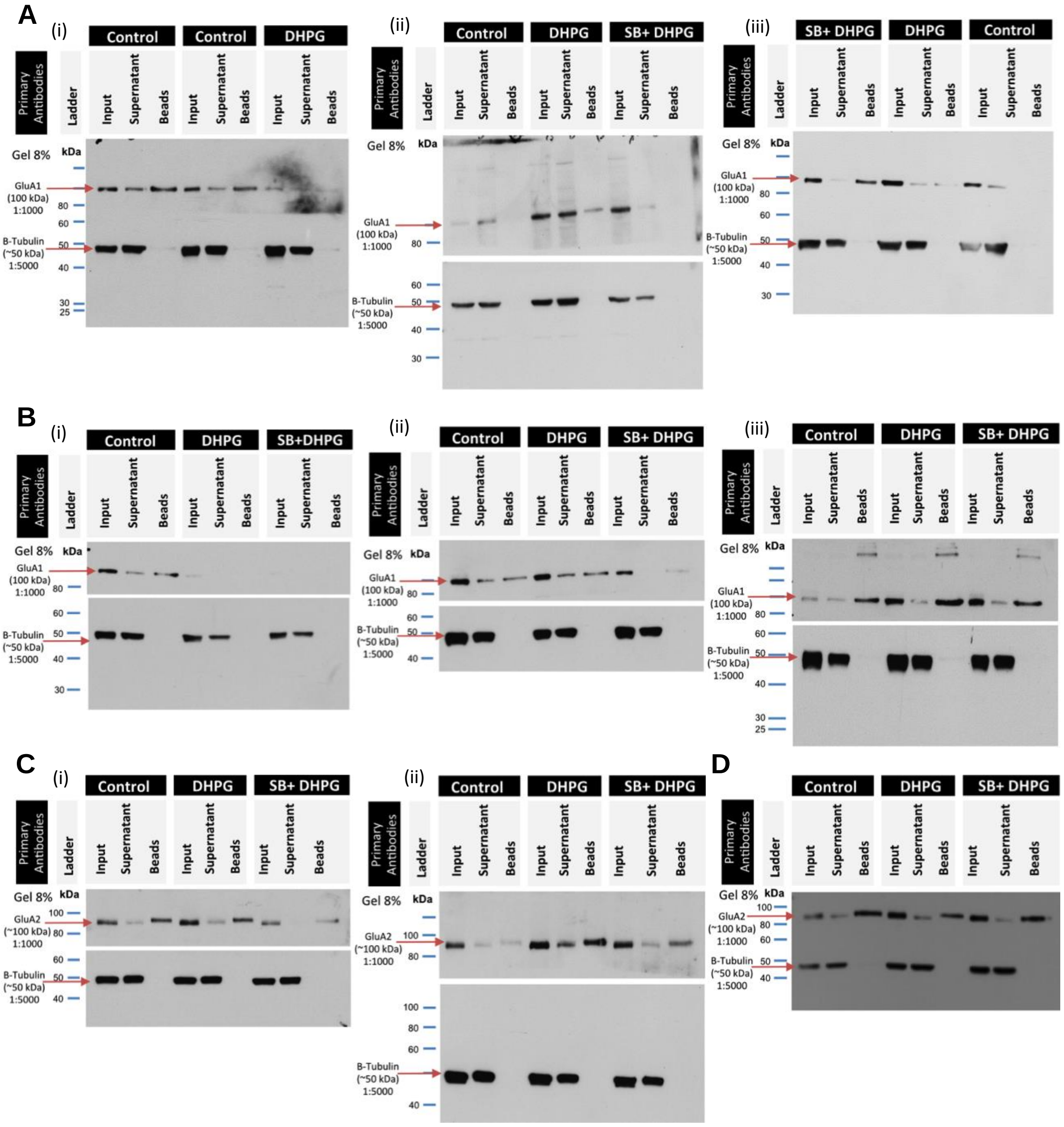

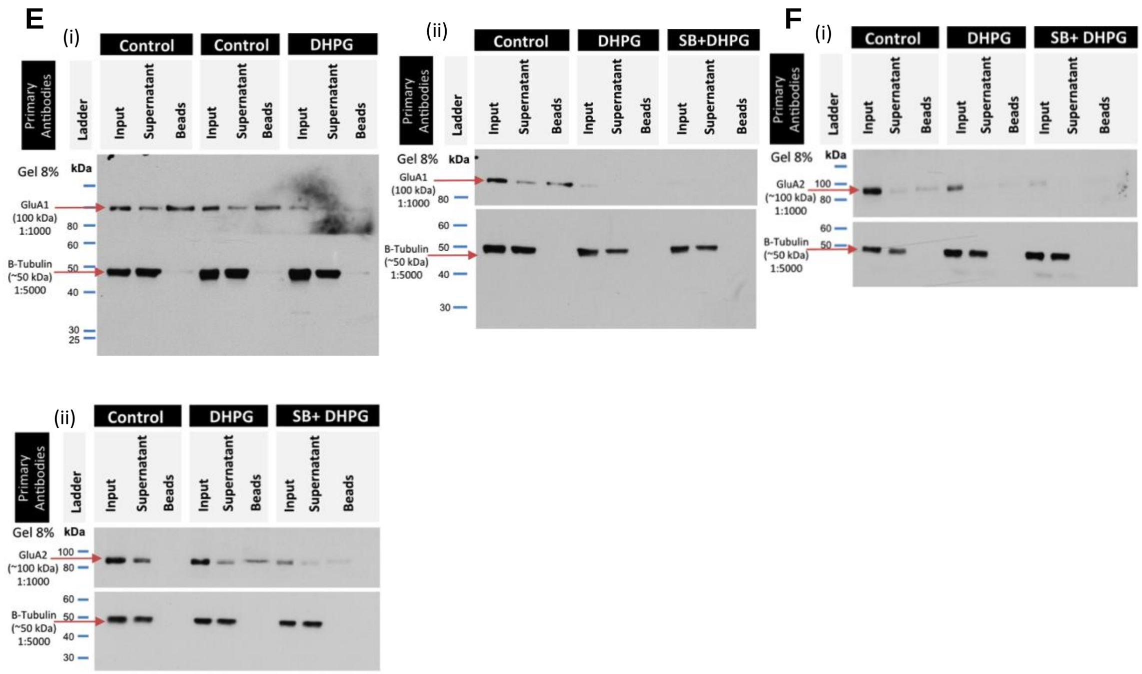

<u>Supplementary figure 1</u>: A) Whole blots from figure 4A WT GluA1 biotinylation, control (i), DHPG (ii) and SB+DHPG (iii) conditions. B) Whole blots from figure 4B DKO GluA1 biotinylation, control (i), DHPG (ii) and SB+DHPG (iii) conditions. C) Whole blots from figure 4C, WT GluA2 biotinylation, control (i), DHPG (i) and SB+DHPG (ii) conditions. D) Whole blot from figure 4D, DKO GluA2 biotinylation, control, DHPG and SB+DHPG conditions. E) Whole blot from figure 4F, WT total GluA1 (i) and DKO total GluA1 (ii). G) Whole blot from figure 4G, WT total GluA2 (i) and DKO total GluA2 (ii).

Supplementary figure 2: The p38-MK2/3-cofilin1 cascade is required in DHPG-LTD (related to Figure 5).

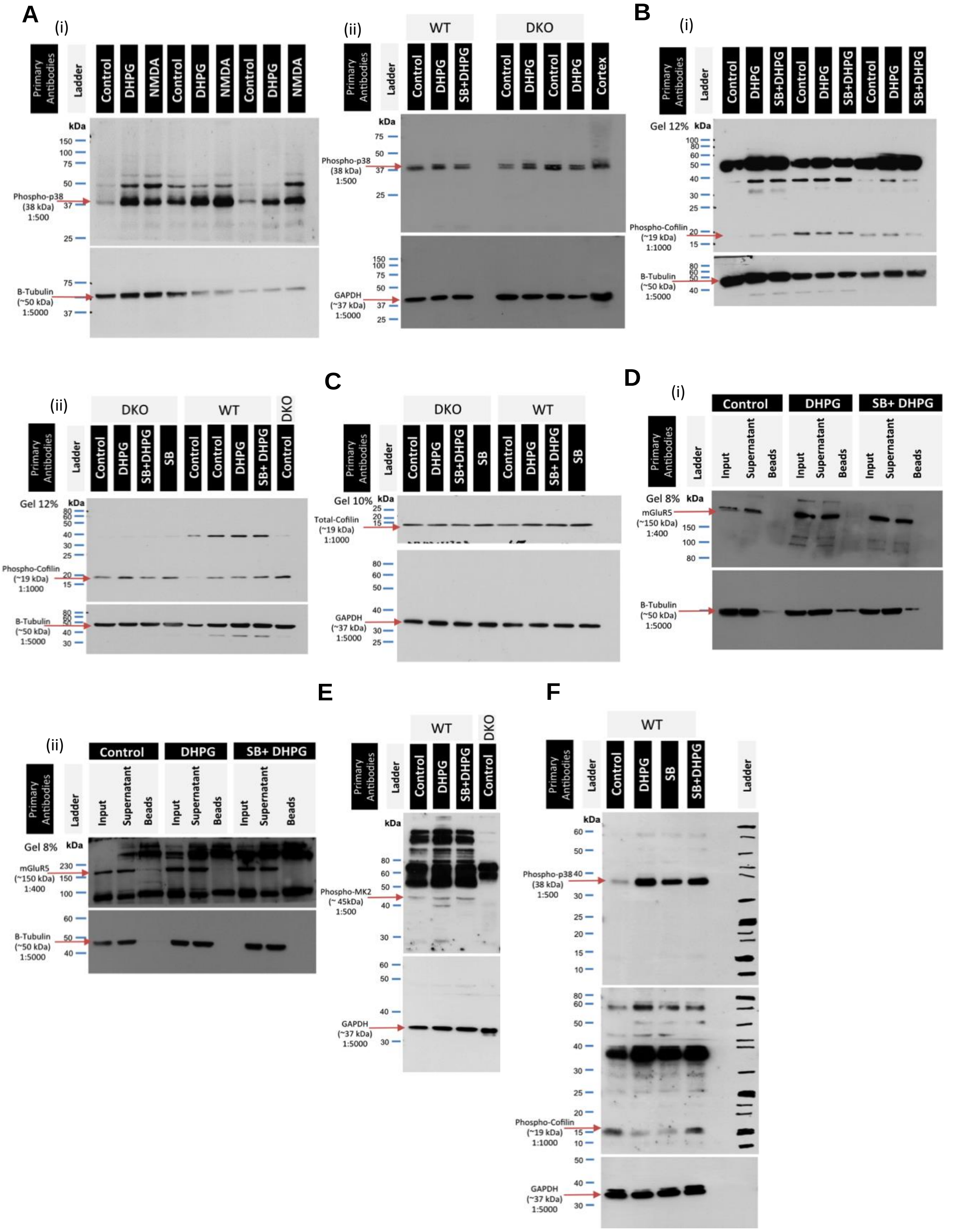

Supplementary figure 2: A) Whole blots from figure 5A, WT p-p38 (i) and DKO p-p38 (ii). B) Whole blots from figure 5B, WT p-cofilin (i) and DKO p-cofilin (ii). C) Whole blot from figure 5C, WT and DKO total cofilin. D) Whole blot from figure 5D, WT (i) and DKO (ii) total mGluR5. E) Whole blot from figure 5E, WT p-MK2. F) Whole blot from figure 5F, p-p38 and p-cofilin.

Supplementary figure 3: MK2/3 DKO mice have deficits in mGluR-LTD (related to Figure 7).

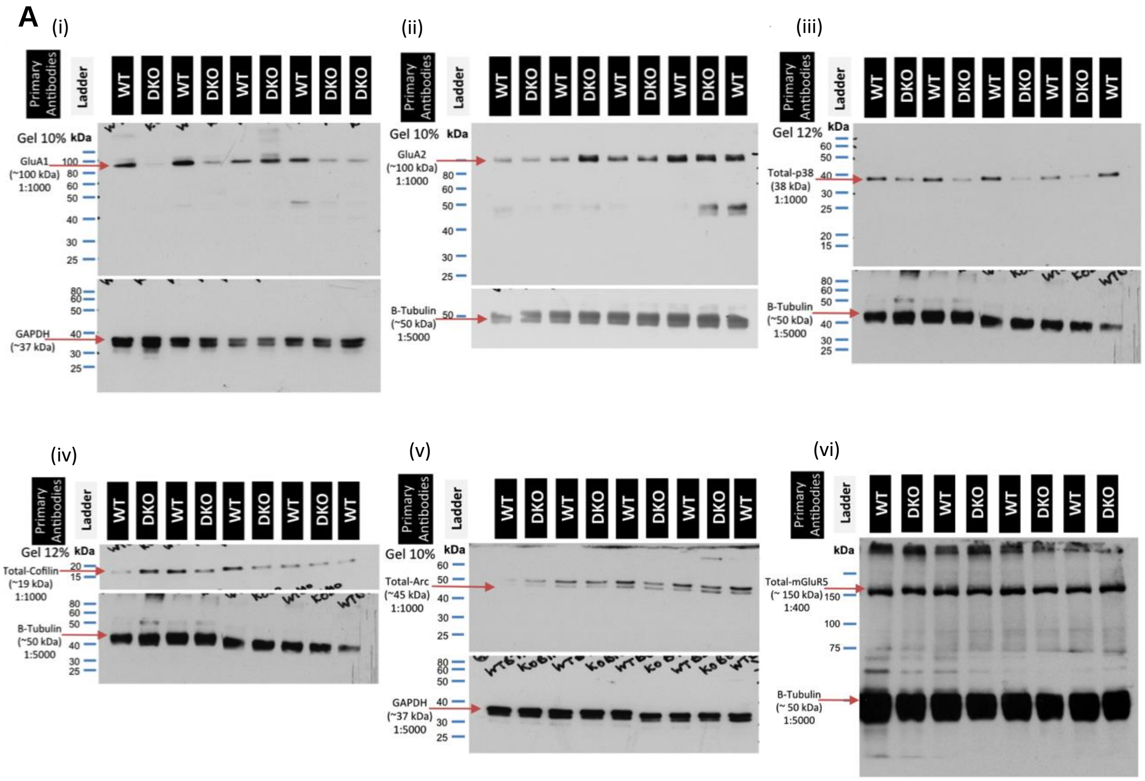

Supplementary figure 3: A) Whole blots from figure 7F, WT and DKO GluA1 (i), GluA2 (ii), Total-p38 (iii), Total-cofilin (iv), Total-Arc (v) and Total m-GluR5 (vi).
